# Mechanical-Stress-Related Epigenetic Regulation of *ZIC1* Transcription Factor in the Etiology of Postmenopausal Osteoporosis

**DOI:** 10.3390/ijms23062957

**Published:** 2022-03-09

**Authors:** Harish K. Datta, Marianne K. Kringen, Stephen P. Tuck, Georgia Salpingidou, Ole K. Olstad, Kaare M. Gautvik, Simon J. Cockell, Vigdis T. Gautvik, Michael Prediger, Jun Jie Wu, Mark A. Birch, Sjur Reppe

**Affiliations:** 1 Musculoskeletal Research Group, Institute of Cellular Medicine, Newcastle University, Framlington Place, Newcastle upon Tyne NE2 4HH, UK; stephen.tuck@nhs.net (S.P.T.); mab218@cam.ac.uk (M.A.B.); 2 Blood Sciences, South Tees Hospitals NHS Foundation Trust, Middlesbrough TS4 3BW, UK; 3 Center for Psychopharmacology, Diakonhjemmet Hospital, 0319 Oslo, Norway; mariannekristiansen.kringen@diakonsyk.no; 4 Department of Engineering, Faculty of Science, Durham University, Durham DH1 3 LE, UK; georgia.salpingidou@durham.ac.uk (G.S.); junjie.wu@durham.ac.uk (J.J.W.); 5 Department of Medical Biochemistry, Oslo University Hospital, 0424 Oslo, Norway; o.k.olstad@medisin.uio.no (O.K.O.); uxresj@ous-hf.no (S.R.); 6 Unger-Vetlesen Institute, Lovisenberg Diaconal Hospital, 0440 Oslo, Norway; karemorten.gautvik@lds.no (K.M.G.); vigdis.gautvik@gmail.com (V.T.G.); 7 School of Biomedical, Nutritional and Sport Sciences, Faculty of Medical Sciences, Newcastle University, Newcastle upon Tyne NE2 4HH, UK; simon.cockell@newcastle.ac.uk; 8 Blood Sciences, The Newcastle upon Tyne Hospitals NHS Foundation Trust, Royal Victoria Infirmary, Newcastle upon Tyne NE2 4HH, UK; mprediger@yahoo.com; 9 Department of Plastic and Reconstructive Surgery, Oslo University Hospital, 0424 Oslo, Norway

**Keywords:** osteoporosis, bone, mechanical stress, epigenetic, *ZIC1* transcription factor

## Abstract

Mechanical loading exerts a profound influence on bone density and architecture, but the exact mechanism is unknown. Our study shows that expression of the neurological transcriptional factor zinc finger of the cerebellum 1 (*ZIC1*) is markedly increased in trabecular bone biopsies in the lumbar spine compared with the iliac crest, skeletal sites of high and low mechanical stress, respectively. Human trabecular bone transcriptome analyses revealed a strong association between *ZIC1* mRNA levels and gene transcripts characteristically associated with osteoblasts, osteocytes and osteoclasts. This supposition is supported by higher *ZIC1* expression in iliac bone biopsies from postmenopausal women with osteoporosis compared with age-matched control subjects, as well as strongly significant inverse correlation between *ZIC1* mRNA levels and BMI-adjusted bone mineral density (BMD) (Z-score). *ZIC1* promoter methylation was decreased in mechanically loaded vertebral bone compared to unloaded normal iliac bone, and its mRNA levels correlated inversely with *ZIC1* promoter methylation, thus linking mechanical stress to epigenetic control of gene expression. The findings were corroborated in cultures of rat osteoblast progenitors and osteoblast-like cells. This study demonstrates for the first time how skeletal epigenetic changes that are affected by mechanical forces give rise to marked alteration in bone cell transcriptional activity and translate to human bone pathophysiology.

## 1. Introduction

Bone is a metabolically active and dynamic organ that remodels throughout life by coordinated activities of bone-resorbing osteoclasts and bone-forming osteoblasts. Mechanical loading plays an important role in the maintenance of bone density, but knowledge of the cellular and molecular biology of mechanosensing remains limited. Mature bone is removed by remodeling that occurs as an adaptive response to different factors, including mechanical loading and replacement of damaged bone following micro- and macrofractures. Studies [1,2] have uncovered the initial molecular mechanisms of *ZIC1* osteocyte actions and implicated a role in human bone pathophysiology. Previously, *ZIC1* had been related to craniofacial developmental defects [3]. Its function was further assessed in a large-scale GWAS meta-analysis focusing specifically on BMD variation in the skull, finding 4 novel loci among 59 with genome-wide significance, including *ZIC1* [4]. The gene was further screened in zebrafish crispants that displayed low skull BMD and abnormal suture patterning [4].

Both osteogenic cell types, osteoblasts and osteocytes, act as mechanosensors and transduce mechanical loading forces into inter- and intracellular biochemical signals [5]. Osteocytes, the most abundant cell type, have many unique characteristics that make them ideal mechanosensors [6,7,8]. In addition to their large number and special distribution in bone, the cells show special morphological features and cell–cell connectivity with each other and several other types of bone cells [7]. The molecular details of how external mechanical load is transmitted to individual cells and specifically to osteocytes have not yet been established. Furthermore, how mechanical loading contributes to the etiology of postmenopausal osteoporosis, a condition of increased bone resorption and fragile bones, is not yet fully elucidated.

The relative significance and contribution of the force, arising from fluid and tissue tethering, in transmitting macroscopic mechanical loading to the microscopic cellular level is uncertain. In addition, mechanical forces acting upon bone generate electrical potentials [9], but their possible physiological importance in influencing the activity of bone cells is unknown.

Skeletal cells, osteoblasts and especially osteocytes respond to mechanical loading by producing a variety of cascading biochemical signals, a process whereby mechanical stress is translated to a biological response [5,10,11]. Mechanically activated osteocytes and osteoblasts modulate several signaling systems; among these, the Wnt/β-catenin pathway clearly plays a powerful role in the regulation of bone mass and new bone formation [12,13]. Previously, it was reported that the zinc finger protein of the cerebellum (*ZIC1*) may mediate fluid-flow-shear-stress-induced modulation by activating the TCF/LEF transcription factor in cell culture [1]. ZIC proteins have been shown to be potential modulators of the hedgehog-mediated signaling pathway and can interact with all GLI proteins, including the repressive form. GLI proteins function downstream of the hedgehog signaling pathway and act as both transcriptional activators and repressors [14,15]. How mechanical loading influences the epigenetic and genetic mechanisms and its downstream effect on the skeletal cell transcriptome and protein translation is, however, not known.

This study investigated whether modulation of the *ZIC1* transcription factor at skeletal sites of high stress in postmenopausal healthy and osteoporotic women was accompanied by DNA methylation changes measured in trabecular bone biopsies. *ZIC1* promoter methylation was significantly lower in bone taken from the lumbar spine compared to the relatively less mechanically stressed os ilium. The extent of DNA methylation of *ZIC1* was linearly negatively correlated with *ZIC1* expression. *ZIC1* mRNA levels in biopsies from the iliac of postmenopausal women showed significant inverse correlation with BMD and were significantly higher in women with osteoporosis than in healthy controls. *ZIC1* promoter methylation was also correlated with *ZIC1* mRNA levels in bone cell cultures, which corroborates its intracellular role in mechanosensing and bone turnover.

## 2. Results

### 2.1. Ex Vivo Studies

#### 2.1.1. *ZIC1* mRNA Expression and Promoter Methylation Differences in Men

**Figure 1:**
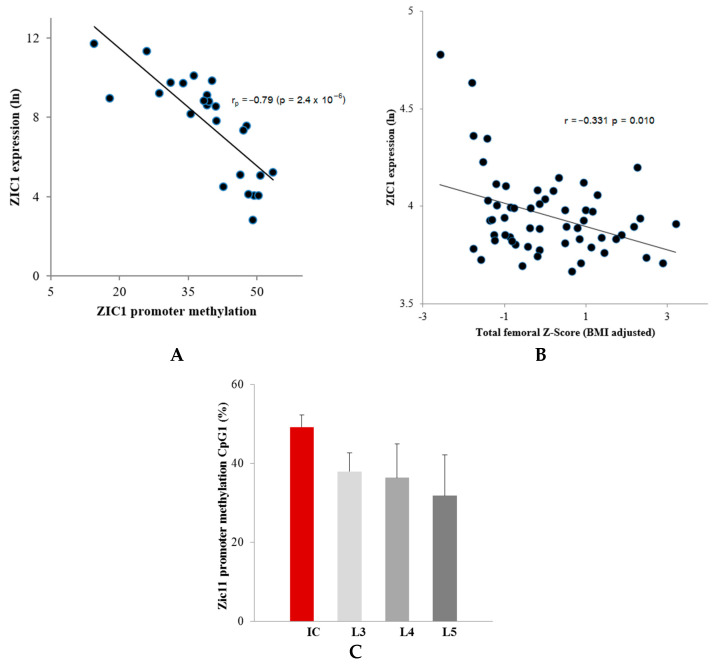
Correlation observed between *ZIC1* promotor methylation and *ZIC1* expression. (**A**) Correlation between *ZIC1* promotor methylation in CpG1 and *ZIC1* expression in the male lumbar spine trabecular bone (Pearson *r* = −0.79, *p* = 2.4 × 10^−6^) (Table 1). (**B**) *ZIC1* expression in the iliac bone of postmenopausal women showed a significant negative correlation with BMD. An inverse association between *ZIC1* expression and total femoral BMD Z-score that had been adjusted for variation in body mass index (BMI) (*r* = −0.383; *p* = 0.0033) was observed. (**C**) A difference was observed in the methylation levels of *ZIC1* promoter at CpG1 from iliac (IB) and lumbar regions (L3–L5) (expressed as %), skeletal sites of low and high mechanical stress, respectively.

Previous initial transcriptome analysis compared gene expression in male human bone biopsy samples taken from lumbar spine (LS) and iliac bone (IB) sites that experience high and low levels of mechanical stress, respectively [2]. These studies showed that the *ZIC1* transcript was profoundly upregulated in the trabecular biopsies from the lamina of the L5 LS vertebrae (77-fold; *p* < 10^−^^9^) in comparison with IB. The present study shows that the degree of DNA methylation also differs markedly between mechanically stressed LS and unstressed IB. The question as to whether *ZIC1* promoter methylation correlated with *ZIC1* expression at the two skeletal sites was addressed using male bone biopsies from LS and IB (Supplementary Table S1). Even if the degree of methylation varied between the denoted positions, CpG1–CpG20, there was nevertheless a significantly higher degree of methylation in the trabecular biopsies from the IB than from LS (Table 1A). Male trabecular bone *ZIC1* mRNA levels and the degree of promoter methylation at positions CpG1 to CpG5, showed a strong negative correlation (Table 1A, Figure 1A). The most intensely methylated promoter region (CpG1) had a 55% higher degree of methylation in IB compared to LS vertebrae (49.2 vs. 31.8; *p* < 0.001) (Figure 1C). All but one (CpG 19) of the 20 CpG methylation sites in the *ZIC1* promoter region showed strong significant inverse correlation between the degree of methylation and *ZIC1* expression (Pearson *r* = −0.33 to −0.74, *p* = 9 × 10^−4^ to 3 × 10^−3^) (Table 1A). Furthermore, *ZIC1* mRNA levels and degree of DNA methylation in each of 20 promoter CpG sites correlate strongly (*r* = 0.72, *p* = 0.0009 for the CpG site in position 1 (CpG1)) (Table 1A).

Interestingly, of the three Affymetrix HU133plus2.0 microarray probe sets for *ZIC1* array expression profile, (236896_at, 234716_at and 206373_at), the probe set 206373_at showed the most variability between IB and LS (Table 1B). The same probe set showed differential expression between osteoporotic and control samples and revealed an inverse association between *ZIC1* expression and total femoral Z-score adjusted for BMI (Figure 1B).

#### 2.1.2. Relationship between *ZIC1* Bone DNA Methylation, mRNA Levels and Postmenopausal Osteoporosis

*ZIC1* expression determined by Affymetrix microarray and PCR analysis in the iliac bone of postmenopausal women with a range of BMD showed a markedly significant inverse correlation with the total BMD adjusted for BMI. A negative association between *ZIC1* expression was seen with total body BMD Z-score that had been adjusted for body mass index (BMI) (*r* = −0.398; *p* = 0.003) and of BMI-adjusted total femoral BMD (*r* = −0.383; *p* = 0.003) and femoral neck BMD (*r* = −0.331; *p* = 0.010) (Table 2, Figure 1B). The demographic and other relevant data for the cohort are given in Supplementary Table S2. There was also a significant inverse correlation with the adjusted lumbar spine L1–L4 BMD (*r* = −0.337; *p* = 0.010). However, no significant correlation was seen with lean body or fat mass (Table 2). Further refined analysis compared *ZIC1* expression in postmenopausal osteoporotics (*n* = 23), defined as T-score ≤ −2.5 at the lumbar spine or hip region with at least one fragility fracture, and age balanced female controls (*n* = 30) with T-score ≥ −0.3), and showed significantly lower *ZIC1* mRNA levels in these patients (Mean ± SD 3.88 ± 0.14 vs. 4.03 ± 0.26, *p* = 0.008).

In view of the increased mRNA expression of *ZIC1* in stressed and high-turnover bone, a possible difference in *ZIC1* DNA methylation, as well as an association between *ZIC1* expression, BMD and osteoporosis was sought in a previously characterised cohort comprised of Norwegian postmenopausal osteoporotic women (*n* = 40) and healthy control subjects (*n* = 29) (Supplementary Table S3) [16,17]. Interestingly, significant differences were observed in CpG methylation levels between osteoporotic (OP) and healthy subjects, as well as after adjustment for multiple testing (Table 3).

#### 2.1.3. Correlation of *ZIC1* Expression with Key Bone-Related Transcripts

**Figure 2:**
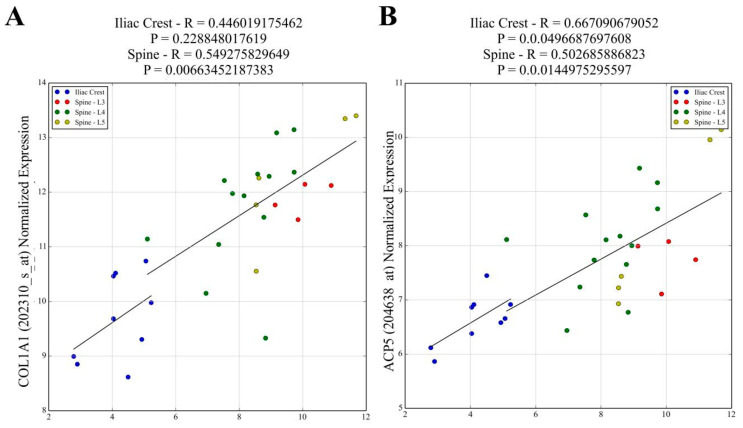

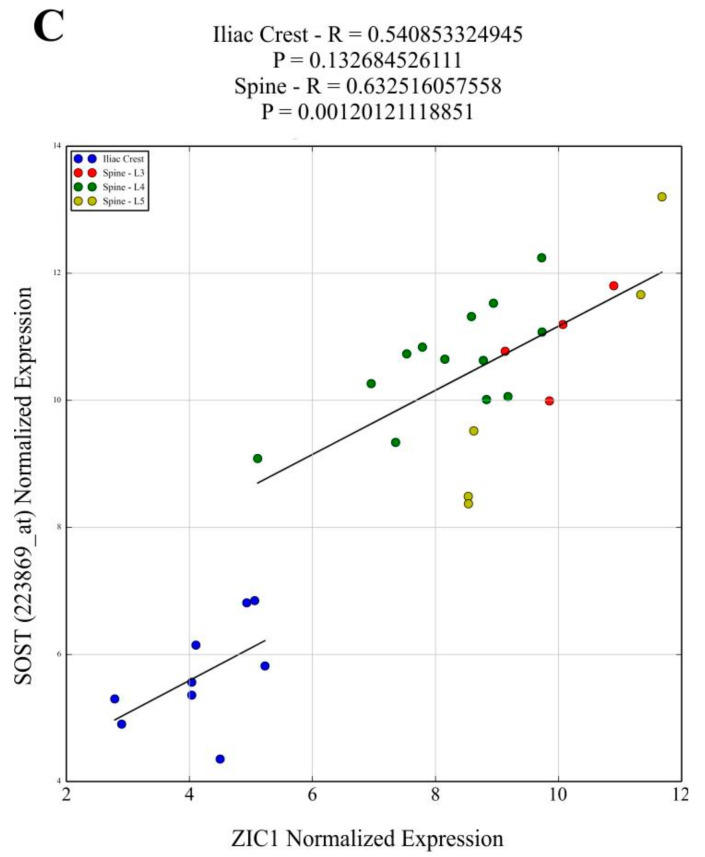
Affymetrix *ZIC1* signal values were correlated with signal values for the transcripts reflecting osteoblast matrix-forming activity. Transcripts for (**A**) *COL1A1*, IBSP*, SPARC*, BGLAP*, CDH11* and osteoblast differentiation (BMP2* and RUNX2*) showed strong positive correlation with *ZIC1* in the lumbar spine but had weaker or no correlation in IB. The PTHR1* transcript used to indicate osteoblast number also showed a strong positive correlation with *ZIC1* expression. (**B**) *ACP5* and CTSK** transcripts that reflect osteoclast activity showed strong positive correlation with *ZIC1* expression. Osteoclast-associated transcripts CALCR** and OSCAR** showed inverse correlation with *ZIC1* expression in the lumbar spine but weaker and no correlation, respectively, in the iliacus, probably due to fewer samples. The expression of osteocyte-associated transcripts (**C**) *SOST*, PDPN*** and MEPE*** at both high (LS) and low (IB) bone turnover sites showed strong correlation with *ZIC1* mRNA. (Please see Supplementary Figure S1. A*, B** and C*** for correlations).

The correlation between the key bone-related transcripts and *ZIC1* expression in loaded trabecular bone (LS) compared to unloaded (IB) bone from male subjects was investigated (Supplementary Table S1), selecting bone-cell-characteristic or widely employed markers of osteoblasts, osteoclasts and osteocytes. The selected transcripts reflecting osteoblast matrix-forming activities were *COL1A1*, *IBSP*, *SPARC*, *BGLAP* and *CDH11* (Figure 2A and Supplementary Figure S1A) and, as makers for osteoblast differentiation, *BMP2* and *RUNX2* (Supplementary Figure S1). These transcripts showed strong positive correlation with *ZIC1* in LS but weaker or no correlation in IB (Figure 2A and Supplementary Figure S1A). The *PTHR1* transcript, used to indicate osteoblast number, also showed a strong positive correlation with *ZIC1* expression (Supplementary Figure S1A). In contrast, transcripts related to osteoclast numbers, such as *CALCR* and *OSCAR*, had significant negative correlation with *ZIC1* expression in the LS but weaker or no correlation, respectively, in the IB, notably due to fewer samples (Supplementary Figure S1B). On the other hand, the transcripts reflecting osteoclast activity, *ACP5* and *CTSK*, showed strong positive correlation with *ZIC1* expression (Figure 2B and Supplementary Figure S1B). These observations are consistent with increased *ZIC1* expression being positively associated with both enhanced osteoblast number and increased cellular activity, whereas its relation to osteoclast activity and number is inverse and appears more complex. Although osteoclast activity was elevated, as reflected by positive correlation with *ACP5* and *CTSK* transcripts, the actual number of osteoclasts, as reflected by *CALCR* and *OSCAR* transcripts, was suppressed (Supplementary Figure S1B). The osteoclastic marker *CALCR* also showed a significant inverse correlation to *ZIC1* in LS (*r* = −0.54, *p* = 0.008) but, again, with no association in IB. Expression of *PDPN*, *MEPE* and *SOST,* osteocyte characteristic transcripts, showed strong correlation with *ZIC1* mRNA at both high (LS) and low (IB) bone turnover sites (Figure 2C and Supplementary Figure S1C). 

### 2.2. In Vitro Studies

#### 2.2.1. *ZIC1* Induction by Fluid Shear Stress in Human SaOS2 Cells and Human Primary Skin Fibroblasts (HSF)

Further in vitro studies were conducted to determine possible association between the degree of *ZIC1* promoter methylation and *ZIC1* mRNA expression. For this purpose, a rapidly proliferating osteoblast-like human osteosarcoma cell line (SAOS2) was subjected to 24 h oscillatory fluid flow, resulting in fluid shear stress (FSS). Basal *ZIC1* mRNA expression levels in SAO2 cells, as determined by qRT-PCR, was found to be 11.4-fold higher than in HSFs (ΔCT 6.98 vs. 10.39). The differences in *ZIC1* expression closely mirrored the degree of *ZIC* promoter methylation in the two cell types, which was considerably higher in SaOS2 cells than in fibroblasts (Table 4A,B), thereby indicating an important regulatory role of promoter methylation in *ZIC1* transcription. When SAOS2 were subjected to 24 h of FSS, there was a significant further rise in *ZIC1* expression, which could be demonstrated despite relatively high pre-existing basal expression (Table 4B). The FSS-induced induction of *ZIC1* in SAOS2 cells was accompanied by an increased expression of ciliary protein KIF3A, which is a subunit of the kinesin II intraflagellar transport (IFT) protein belonging to the kinesin-2 family, and of *SP7* transcription factor (Osterix) (Table 4B). In contrast to SAOS2 cells, in HSF cells, exposure to fluid shear stress failed to induce a significant increase in *ZIC1* expression despite the low basal expression (Table 4C). However, neither cell type responded with statistically significant changes in the degree of *ZIC1* promotor methylation.

#### 2.2.2. Induction of *ZIC1* mRNA by FSS in Rat Osteoprogenitor Cells

**Figure 3:**
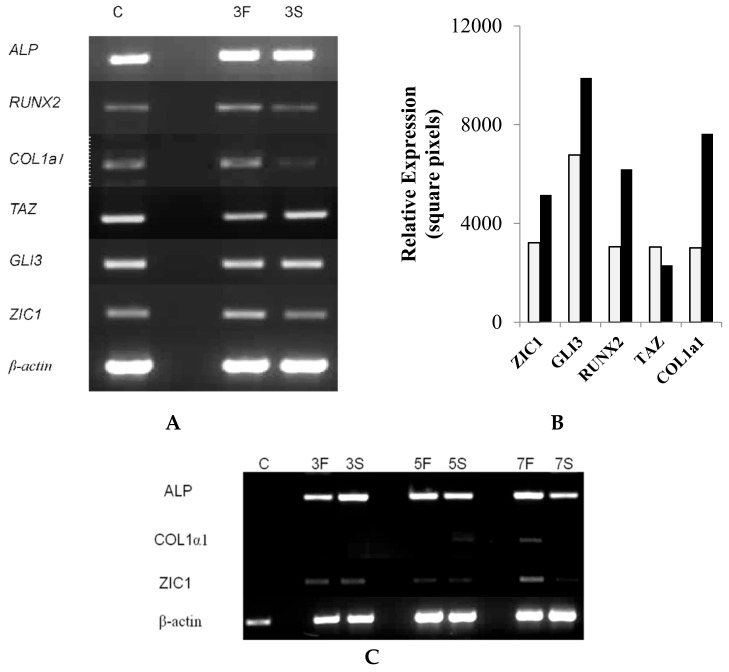
The effect of fluid shear stress (FSS) on osteogenic genes. (**A**) Rat osteoprogenitor cells were cultured in osteogenic medium and subjected to FSS (F). Control cells are labelled S (stationary). The cells subjected to FSS for three days (lane 3F) had induction of the key osteogenic marker genes Runx2, ALP and Col1a1 when compared to controls (lane C). (**B**) The induction was confirmed by qRT-PCR in the FSS and control stationary cells. (**C**) Cells cultured in normal non-osteogenic medium, subjected to similar magnitude of FSS, showed no evidence of *Zic1* or *Col1a1* induction over three days (lane 3F vs. 3S). However, after prolonged shear stress, there was an induction of *Col1a1*, as well as *ZIC1*, in these cells (lanes 5F, 5S, 7F and 7S).

Further in vitro investigations were designed to elucidate the role of *ZIC1* in the differentiation of osteoblasts from rat osteoprogenitor cells (ROPs) and to determine whether mechanical stresses influence this process. ROPs were cultured in osteogenic medium and subjected to FSS for three days, followed by qRT-PCR analyses of osteogenic markers and *Zic1*. The results showed that FSS led to induction of the key osteogenic markers *Runx2, Alpl* and *Col1a1* when compared to controls (Figure 3A,B). However, the mRNA level of *Taz1*, an important marker of osteogenic potential, was unaltered (Figure 3A,B). When the rat osteoprogenitor cells were cultured in normal medium but subjected to similar magnitude of FSS, no induction of *Zic1* or *Col1a1* was seen over three days (Figure 3C). However, a further exposure of these cells to FSS in normal medium led to induction of both *Col1a1* and *Zic1* (Figure 3C).

#### 2.2.3. Immunocytochemical Localization of Rat *ZIC1* Protein

**Figure 4:**
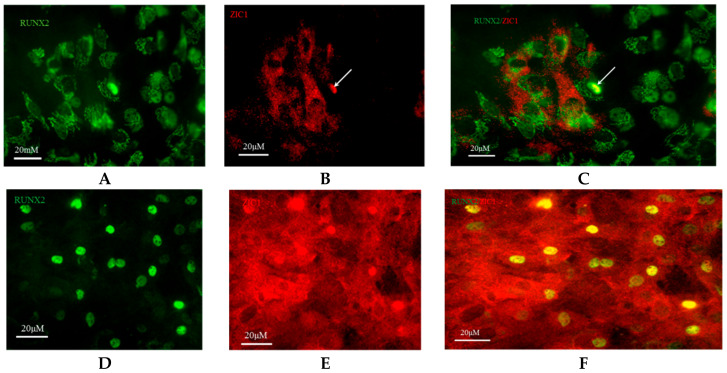
Rat osteoprogenitors (ROPs) cultured in osteogenic medium and maintained as static controls were fixed on 25 mm coverslips and immunostained at the end of day 3 with (**A**) anti-RUNX2 (green fluorescence, plate 1) and (**B**) anti-*ZIC1* (red fluorescence, plate 2) antibodies. (**C**) Plates 1 and 2 were merged together to identify co-localisation of *RUNX2* and *ZIC1* antibodies (Plate 3). In the representative field of vision, many nuclei showed nuclear translocation of *RUNX2*, suggesting relevant osteogenic activity in these cells. A few nuclei showed co-localisation of *RUNX2* and *ZIC1,* and it appears that expression of *RUNX2* (yellow) was accompanied by some *ZIC1* (red/green). The effect of FSS on the intracellular distribution of *RUNX2* and *ZIC1* in ROPs is seen in (**D**) plate 4 and (**E**) plate 5, respectively. (**F**) Plates 4 and 5 were merged together to identify co-localisation of *RUNX2* and *ZIC1* antibodies (Plate 6).

**Figure 5:**
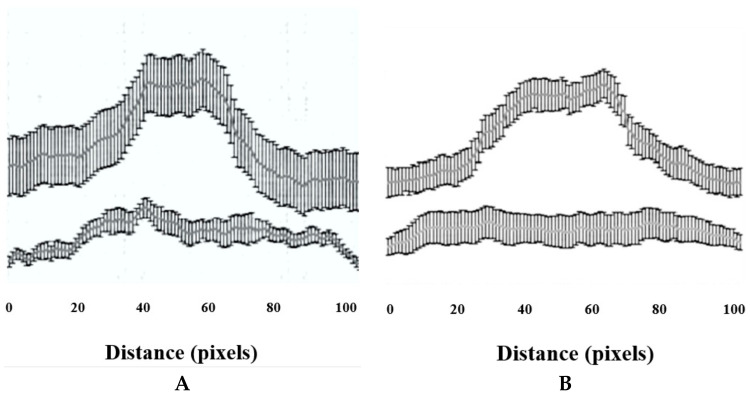

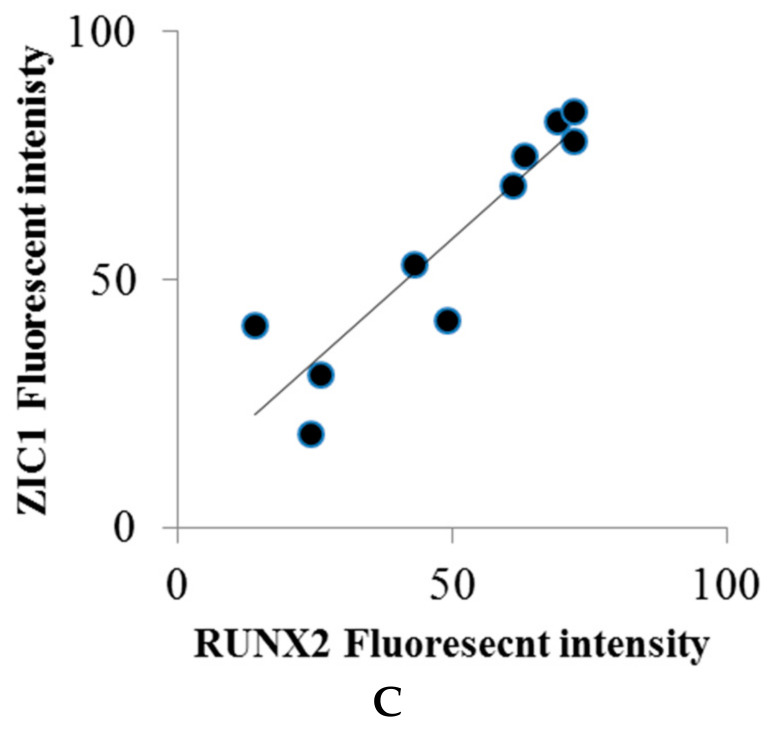
(**A**,**B**) The fluorescence intensity of *RUNX2* and *ZIC1* nuclei (expressed as mean, grey values) from ROPs cultured in osteogenic medium, maintained either as static control or subjected to FSS at the end of day 3. (**C**) The plot shows a direct relationship between translocation of *RUNX2* and *ZIC1* to the nucleus when subjected to FSS.

Immunocytochemical localization of *ZIC1* protein in rat osteoprogenitor cells cultured in osteogenic medium showed greater overall concentration of *ZIC1* protein in rat osteoprogenitor cells subjected to FSS than static controls (Figure 4 and Figure 5), mirroring the increased expression of *Zic1* mRNA (Figure 3). In static control cells, *RUNX2* showed considerable perinuclear staining, with a proportion of cells lacking *RUNX2* in the nucleus (Figure 4 and Figure 5). The fluorescence intensity of both *RUNX2* and *ZIC1* in the cytoplasm and nuclei of cells subjected to FSS compared to static controls showed increased overall protein expression, as well as evident translocation from cytoplasm to nuclei (Figure 4A–F). A plot of the intensity of *ZIC1* and *RUNX2* fluorescence in nuclei of cells subjected to FSS and static cells shows clearly that the nuclei of cells subjected to FSS reveal greater nuclear translocation of and *RUNX2* (Figure 5A,B). The graph shows a linear relationship, suggesting a direct relationship between translocation of *ZIC1* and RUNX2 in the nucleus after application of FSS (Figure 5C).

## 3. Discussion

It is generally accepted that external loading influences bone cells in two main ways: as a shear force and through cellular deformation [18,19]. The bone matrix, a heterogeneous structure, may act as a stress conductor and amplify the strain experienced by the osteocytes by as much as 15-fold compared to that experienced by the macroscopic bone [18,19,20].

We therefore studied bone biopsies taken from high or low mechanical loading sites representing elevated and suppressed bone turnover, respectively, and found a close negative correlation between *ZIC1* promoter methylation and gene expression. At the site of high loading and increased bone turnover, *ZIC1* mRNA expression was elevated, and *ZIC1* promoter methylation was reduced. In contrast, in the iliac bone, a site of low mechanical loading, *ZIC1* mRNA expression was substantially lower. A strong correlation between *ZIC1* expression and transcripts characteristically expressed in osteoblasts, osteocytes and osteoclasts was documented. We then investigated a possible association between the transcription factor and osteoporosis and BMD in transiliac bone biopsies from postmenopausal Norwegian women and found a negative correlation between total, spinal and hip BMD Z-scores, respectively, and *ZIC1* expression. The correlation was even stronger when BMD was adjusted for BMI variation. Therefore, this is the first study to link skeletal epigenetic changes with mechanical stresses and associated increase in bone turnover. The study also demonstrates an association between *ZIC1* expression, the degree of *ZIC1* promoter methylation, bone turnover and BMD.

To corroborate ex vivo observations in human bone biopsies, we studied the effects of mechanical stresses (FSS) on the modulation of key osteogenic genes, *ZIC1* mRNA expression and its promoter methylation in cultured cells. We asked whether mechanical stress could upregulate *ZIC1* mRNA in isolated rat osteoprogenitor cells, whcih possess osteogenic potential and affect the osteogenic response. The transcriptional response of the cells to FSS in a two-armed study (mechanically strained vs. static cells) was carried out to rule out factors other than FSS affecting the osteogenic response of the cells. Following the application of mechanical strain, the expression of genes associated with osteogenic differentiation (*Alp*, *Runx2* and *Col1a1*) and genes of interest that might play a role in mediating the effect of FSS (*Taz*, *Gli3* and *Zic1*) were determined (Figure 3A–C). Immunocytochemical localization of *ZIC1* and *RUNX2* in rat osteoprogenitor cells subjected to FSS was further compared with controls and showed increased *ZIC1* and *RUNX2* levels. Intense staining for *RUNX2* was found in the nuclei, and we also demonstrated a significant *ZIC1* co-localisation (Figure 4A–F). The fluorescent intensity of *ZIC1* and *RUNX2* in the cells was found to be significantly positively correlated (Figure 5C). A comparative analysis of the expression of osteogenic marker genes and *Zic1* in rat osteoprogenitor cells subjected to FSS with static controls suggested that FSS was essential for the induction, promotion and maintenance of an osteogenic phenotype in ROPs (rat osteoprogenitors). Furthermore, FSS leads *to ZIC* mRNA induction.

It was notable that *ZIC1* promoter methylation was markedly distinct at 20 CpG sites between loaded LS and unloaded IB bone and presented a strong negative correlation with *ZIC1* mRNA levels (Table 1A,B) (Figure 1A). The expression of *ZIC1* was also strongly inversely correlated with age and BMI-adjusted BMD in postmenopausal women. This may be explained by *ZIC1* as an important sensor molecule in coupling bone stress to biological responses regulating bone remodeling. *ZIC1* may respond to increased load with enhanced gene transcription. Osteocytes/osteoblasts in osteoporotic subjects (OP) with thinner and fewer trabeculae endure enhanced load and possibly microfractures and respond to this by increased *ZIC1* transcriptional activity, possibly as a result of hypo methylation. We attempted to corroborate this hypothesis with our experiments using osteogenic cell cultures.

The importance and relevance of DNA methylation of genes involved in bone metabolism are underscored by several papers demonstrating associations. For example, we have previously shown that DNA methylation of *SOST* in bone is associated with fracture risk PMID: 25155887. Additionally, as reviewed PMID: 33569383, PMID: 33921902 several osteogenic differentiation markers and genes involved in bone metabolism have been shown to be regulated by DNA methylation, including *RUNX2*, *SP7*(*OSX*), *ALP*, *RANKL*, and *OPG*.

It has been previously shown that a shear-stress-induced increase in T-cell factor/lymphoid enhancer factor transcriptional activity in osteogenic cells was abolished by *ZIC1* silencing, implying that *ZIC1* has an important role in shear flow mechanotransduction [1]. In order to establish whether observed changes in *ZIC1* epigenetic promoter and accompanying increased *ZIC1* mRNA expression were causally linked to mechanical loading, we subjected rat osteogenic cells and human osteoblast-like cells (SAOS2) to FSS.

SAOS2 and HSF cells were used to study *ZIC1* promotor methylation before and after 12 h of fluid shear stress, conditions proven to activate the osteogenic response, but induced no statistical differences in either cell type. For the reasons unknown, the degree of basal *ZIC1* promoter methylation differed markedly between the two cell types, as did *ZIC1* expression. It is possible that FSS, over hours, may not represent a suitable in vitro model for DNA methylation caused by inductive stress. However, in vivo bone anabolic response can never be fully mimicked in in vitro cell culture experiments. This is particularly the case when it involves regulation of complex signal–transductions reactions as initiated by *ZIC1* activation of osteocytes located in bone lacunae.

Despite considerable progress in identifying mechanosensing and mechanotransduction-signaling molecules and pathways, there is a lack of knowledge as to how the transmitted information from the membrane is converted into transcriptional changes. An earlier comparison [2] of global gene expression using microarrays (human Affymetrix gene chip) in human bone samples from sites that experience high and low levels of mechanical stress, lumbar spine and iliac crest, respectively, revealed that *ZIC1* was significantly upregulated in the lumbar spine compared to the iliac crest. More recently, we showed that that the zinc finger protein of the cerebellum (*ZIC1*) plays an important role in shear flow mechanotransduction in osteocytes and osteoblasts, as well as activation of the primary cilia to mechanotransduction in bone [1]. Therefore, we investigated whether stress-related increased expression of *ZIC1* was accompanied by epigenetic changes by comparing *ZIC1* promoter DNA methylation in male iliac crest and lumbar spine. Trabecular bone was used, as it has an extensive surface representing the main site of bone metabolic activity. *ZIC1* promoter methylation was significantly lower in the lumbar spine than in the iliac crest, and the extent of DNA demethylation was linearly negatively correlated with *ZIC1* gene expression (Table 1A, Figure 1A).

The *ZIC1* expression in human trabecular bone was positively correlated with osteoinductive bone morphogenetic protein 2 (*BMP2*) (Supplementary Figure S1A). The possible role of ZIC1 in promoting osteogenesis was underlined by its significant positive correlation with transcripts characteristically associated with osteogenic cells, osteocytes (*SOST*, *DKK1* and *PDPN*) and osteoblasts (*COL1A1*, *BGLAP*, *SPP1*, *CDH11* and *PTHR1*) and common to both cell types (*RUNX2*, *IBSP* and *MEPE*) (Figure 2 and Supplementary Figure S1). A few transcripts associated with osteoclast activity showed positive correlation (*CTSK* and *ARC5*) with *ZIC1* expression, whereas *OSCAR* showed inverse correlation (Supplementary Figure S1B). The observations reported in this paper combined with those from earlier work establish that *ZIC1* upregulation at sites of high stress and high bone turnover has an important role in mechanical-stress-induced increases in osteogenic differentiation and osteoblast and osteocyte activity. The present data suggest that this increase in *ZIC1* expression in human trabecular bone was the result of reduced methylation at its promoter region.

Finally, the results also suggest that increased mechanical loading and stress present in the fewer and thinner trabeculae in osteoporotic bone with ensuing reduced *ZIC1* promoter methylation is likely a compensatory mechanism aiming to increase *ZIC1* transcription and bone formation. Additionally, the expression of *SOST*/sclerostin, an important negative regulator of the Wnt receptor anabolic pathway, is compensatory reduced at the mRNA and protein level in osteoporotics, accompanied by hypermethylation of its promotor [21]. Since *ZIC1* expression is increased, whereas *SOST* expression is reduced at low BMD, a positive correlation between *SOST* and *ZIC1* mRNA levels may appear paradoxical. However, the results make sense if both transcripts change their expression as compensatory mechanisms attempting to counteract the bone loss.

## 4. Material and Methods

### 4.1. Male Participants

All participants were Caucasian men (*n* = 13) from the northeast of England, U.K. The vertebral biopsies from these subjects were collected whilst they were undergoing spinal fusions or spinal decompression laminectomies of the lumbar spine (LS). The study was conducted as per ethical principles for medical research involving human subjects expressed in the World Medical Association Declaration of Helsinki. The study was commenced after ethical approval was obtained from the Institutional Review Board of Northumberland Local Research Ethics Committee, Blyth, Northumberland NE24 2AG (REC 09/05/2005; Reference: 04/Q0902/29). Informed written consent was obtained from all the study participants. All possible secondary causes of bone loss were excluded by relevant detailed medical history. Medical examination and a range of laboratory investigation are detailed in Supplementary Materials. Patients with a history of oral glucocorticoids, bone preservation agents, anticoagulants or anticonvulsants were excluded [2]. The mean ± SD (range) of anthropometric indices and BMD, given as areal density, is presented in Supplementary Table S1.

### 4.2. Female Participants

The female participants were a randomly selected subset (*n* = 57) from the ‘Osteogene Study’ cohort comprised of 84 Norwegian women (50–86 years) with a varying range of BMDs who were free of metabolic bone disease. All these subjects were recruited at the outpatient clinic of Lovisenberg Diaconal Hospital, Oslo (Supplementary Table S4).

Transiliac bone biopsies were obtained from ilia at the same location as described previously [16]. The power of the cohort has been previously reported [16,17].

### 4.3. RNA Purification and Gene Expression Analysis

Microarray analysis of total RNA on HG-U133 plus 2.0 chips (Affymetrix Santa Clara, CA, USA) was performed as previously described [2]. TaqMan gene expression analysis (real-time RT-PCR validation) of selected transcripts (*n* = 21) (as per the manufacturer’s instructions), together with the ribosomal protein L41 (RPL41) as internal standard [17], was used to control the reproducibility of the analysis on the chips. The rationale for selecting ribosomal protein L41 (RPL41) as an internal standard was that Affymetrix analyses had shown a similar magnitude of signal in the female bones (10,895 ± SD 315). The relevant female data were submitted to the EMBL-EBI (European Bioinformatics Institute) ArrayExpress repository (accession number: E-MEXP-1618); the male data can be accessed at the same site (accession number: E-MEXP-2219).

### 4.4. Bone Biopsies

To avoid possible anatomical-site-related variability, all bone biopsies were trabecular and were taken from a specific iliac site, namely two centimetres distal to the iliac crest and two centimetres posterior from the anterior superior iliac spine [17]. To avoid interoperator variability, all biopsies were collected under the supervision of one named senior surgeon, who was instructed to avoid attached tissue and muscle.

Likewise, male biopsies from the lumbar spine were also trabecular. These were primarily from the lamina process of lumbar vertebrae (L2, L3 or L4), whereas 19 biopsies from the vertebral lamina of the LS and 5 biopsies were from the iliac crest (IB).

Affymetrix *ZIC1* signal values were correlated with signal values for the transcripts reflecting osteocyte (*SOST*, *PDPN* and *MEPE*), osteoblast (*COL1A1*, *IBSP*, *SPARC*, *BGLAP* and *CDH11*), osteoblast differentiation (*BMP2* and *RUNX2)*, and osteoclast activity (*CALCR* and *OSCAR*).

### 4.5. Global Methylation Analyses

Analysis of the DNA obtained from iliac bone biopsies was performed using an Infinium HumanMethylation450 BeadChip (Illumina) as per the manufacturer’s instructions. This allowed for detection of the methylation status of 485,000 individual CpGs. On average, there were 17 CpG sites per gene region, which were found to be distributed across the first exon, gene body, promoter, 5′UTR and 3′UTR. The quantitative measurement of the methylation for each CpG (β value) was obtained from the fluorescence data in BeadStudio (Illumina). The data were preprocessed using minfi and normalized using BMIQ [22,23].

### 4.6. ZIC1-Specific Methylation Analysis

The methylation pattern of 20 consecutive CpGs within the promoter region of *ZIC1* was measured using pyrosequencing. DNA from female bone biopsies (~100 ng) and/or human osteoblast-like cells (SAOS2) (~1 µg) was bisulphite-treated and purified using an EpiTect bisulphite kit (Qiagen, Germantown, MD, USA). A 207 bp amplicon (chr3:147,126,936–147,127,142; GRCh37/hg19 assembly) was amplified from ~10 ng of bisulfite-treated DNA in 25 µL reactions using 1× PyroMark PCR Master Mix, 1× CoralLoad Concentrate (both from Qiagen) and 0.2 µM primers. Primers were designed using PyroMark Assay design 2.0 (Qiagen); forward primer: 5′-TGGTTTGTTAAAAGGGGATGTT-3′ and biotinylated reverse primer 5′-B-ACACCCTCCCCCCCTTAATAA-3′. Cycling conditions were an initial denaturation step at 95 °C for 15 min, followed by 50 cycles of 94 °C for 30 s, 56 °C for 30 s, 72 °C for 30 s and a final extension period at 72 °C for 10 min.

A total of 20 µL of PCR products was added to 40 µL binding buffer (Qiagen), 2 µL streptavidin–Sepharose high-performance beads (GE Healthcare, Chicago, IL, USA) and 18 µL water and stirred for 5–10 min at 14,000 rpm. Single-stranded biotinylated templates were isolated using PyroMark Vacuum Prep WorkStation (Qiagen) and dispensed onto a PyroMark Q24 plate containing 25 µL of 0.3 µM sequencing primer (5′-AAGAGTTTTATAATATTTGGGATTG-3′) and annealing buffer (Qiagen). The plates were incubated for 2 min at 80 °C and subsequently cooled at room temperature for at least 5 min. Sequencing was performed in a PyroMark Q24 instrument with PyroGold reagents (Qiagen). Results were analysed using PyroMark Q24 2.0.6 software, Qiagen, Germantown, MD, USA.

### 4.7. Data Analysis

Data analysis was performed by importing CEL files (raw gene expression data) into Partek Genomics Suite software (Partek, Inc., St. Louis, MO, USA). Generation of signal values and normalization was performed by the application of the robust multichip analysis (RMA) algorithm. A one-way analysis of variance (ANOVA) model was used for the expression comparisons of different groups. The results are given as fold change.

In accordance with the manufacturer’s recommendation, raw gene expression data were normalized and processed using the Affymetrix GCOS software module and MAS 5.0 in ArrayAssist (Stratagene, La Jolla, CA, USA), which generated a list of genes ‘present’ in a given sample. Following the compilation of a raw data set identification of differentially expressed genes was performed by ArrayAssist software. Statistical significance of microarrays was assessed by unpaired *t*-test, which identified differential gene expression. Normalized data were filtered on expression, with a cutoff value of two-fold change in expression (*p* ≤ 0.05). Gene ontology/enrichment analysis was then performed on the differentially expressed clusters.

### 4.8. Cell Lines and Culture Conditions

Immortalized human SAOS2 osteoblast-like cells (ATCC, Manassas, Virginia, USA) were cultured in 25 cm^2^ flasks and incubated at 37 °C in 5% CO_2_. Briefly, in each experiment, SAOS2 cells were treated with trypsin-EDTA solution (Sigma-Aldrich, Merck KGaA, Darmstadt, Germany), removed from the flask surface and grown in McCoy’s 5a medium (Sigma-Aldrich) supplemented with 10% foetal bovine serum (FBS). For primary human skin fibroblast (HSF) cells, Dulbecco’s modified Eagle’s medium (DMEM, Sigma-Aldrich) supplemented with 5% Calf Serum (CS), 100 μg/mL Streptomycin and 100 U/mL penicillin was used. Both cell lines were being subcultured when they reached 70–80% confluence.

### 4.9. mRNA Extraction and Quantification

Cells were grown in original 25 cm^2^ culture flasks until they reached 80% confluence. Normal medium was removed, and the flasks were washed with PBS to ensure that no residual media or growth factors were present. Cells were then lysed by directly adding 1 mL of Trizol reagent (Invitrogen, Thermo Fisher Scientific, Waltham, MA, USA) in the flask, passed through the pipette several times to homogenize the lysates, transferred to RNAse-free Eppendorf tubes and left for 5 min to completely dissociate the nucleoprotein complexes. An amount of 200 μL of chloroform was then added to the tubes, shaken vigorously for 10–15 s and left for another 3 min. Tubes were then centrifuged at 13,000 rpm for 15 min, and the colourless upper aqueous phase containing RNA was removed from all tubes, transferred to new tubes and precipitated by mixing with an equal volume of isopropyl alcohol. Samples were then left for 10 min, centrifuged at 13,000 rpm for 15 min and supernatant was removed. Remaining pellets were washed once with 1 mL of 70% ethanol, mixed by vortexing and centrifuged at 13,000 rpm for 3 min. Ethanol was removed, and RNA pellets were left to dry completely for 5 min and finally redissolved by adding 50 μL of RNase-free water, passed through the pipette tip several times and incubated for 10 min at 60 °C. The absorbance of each sample was then measured at 260 nm by a spectrophotometer (NanoDrop, Thermo Fisher Scientific, Waltham, MA, USA) to calculate the concentration of mRNA in ng/μL. In addition, calculating the 260/230 nm and 260/280 nm absorbance ratios determined the integrity and purity of extracted RNA. Prior to each measurement, the spectrophotometer was calibrated with a blank (RNase-free water) at 260 nm to provide a more accurate reading.

The first-strand cDNA was synthesized from extracted mRNA by reverse transcription (RT). A total of 4 μg of total RNA was used as starting template for each reaction and included oligo dT, dNTP (10 mM). The volume of water added for each reaction was adjusted depending on the concentration and volume of mRNA added to give a final volume of 14 μL.

### 4.10. Immunofluorescence Detection of ZIC1

Primary rat osteoprogenitor cells were isolated from 3–4-day-old rat pup calvariae. The pups were sprayed and cleaned with 70% alcohol before the calvariae were dissected and washed with balanced Hanks solution, cut into small 2–3 mm wide pieces and digested with 1 mg/mL collagenase (type II) on a rocking bed. The supernatant was discarded, and the collagenase reaction was repeated for a further 30 min, following which the supernatant was isolated and kept as fraction 1. Calvariae were then washed with PBS that was added to fraction 1. In the next step, fraction 2 was collected by washing calvariae with PBS containing 4 mM EDTA for 15 min at 37 °C on a rocking bed. The calvariae were washed once more with Hanks solution and added to fraction 2. The collagenase reaction was repeated for 30 min, and the supernatant was kept as fraction 3. The calvariae were once again washed with Hanks solution, and this was added to fraction 3. In the final step, all the fractions were pooled and spun at 800× *g* (1000 rpm) for 3 min, and the resulting cell populations were cultured in T75 flasks for further experiments. The rat calvarial osteoblasts used were a mixed primary culture that contained cells at a number of points in the continuum from progenitors to mature cells. Previous characterization of the cells showed their response to osteoinduction, with increased levels of alkaline phosphatase and deposition of mineralised matrix, as well as appropriate changes in expression of alkaline phosphatase, collagen I, osteopontin and osteocalcin [24].

Cells were harvested and grown on sterile 13 mm glass cover slips (in a 24-well plate) overnight at 37 °C to let them settle down and adhere on the surface. Then, cells were briefly washed with PBS, and freshly prepared (<3 months) preheated (37 °C) 4% paraformaldehyde in PBS was added for 10 min to fix cells on the surface. Cover slips were thoroughly washed with PBS/0.1% Tween three times, and specimens were then incubated with 3% goat serum dissolved in PBS/0.1% Tween for approximately 30 min. Goat serum was used as a blocking serum to prevent non-specific binding of IgG, as secondary antibody was originally raised in goat. After a brief wash with PBS/0.1% Tween, 150 μL of rabbit polyclonal anti-ZIC1 antibody (diluted at 2.5 μg/mL in PBS, Abcam, Cambridge, UK) was added and incubated at room temperature for 2 h.

After the 2 h incubation time, cover slips were extensively washed with PBS/0.1% Tween three times (each wash for 15 min), and 2.5μL of anti-rabbit IgG (whole molecule, FITC conjugate, Sigma-Aldrich) was added per cover slip (in a well containing 500 μL PBS) and incubated at room temperature for 1 h in a dark chamber. After three extensive washes with PBS/0.1% Tween, cover slips were dried and mounted with Vectashield with DAPI (Vector) on microscope slides and examined on a microscope (DMLB, Leica, Wetzlar, Germany) with a fluorescent lamp (ebq 100 isolated, Leistungselektronik, Jena, Germany).

### 4.11. In Vitro Fluid Shear Stress Studies

To address the question of whether mechanical stress can upregulate *ZIC1* in isolated rat osteoprogenitor cells and if so, whether *ZIC1* was associated with enhanced osteogenesis (rat progenitors), we performed studies on freshly isolated primary rat osteoprogenitor cells. Cells were seeded on sterile 13 mm glass coverslips (in a 24-well plate) or in culture flasks incubated at 37 °C overnight and were then serum-starved for another 12 h. Serum-free medium of the culture flaks was then replaced with 5 ml of normal medium supplemented with 5.958 g/L HEPES and transferred to an orbital shaker incubator (Gallenkamp; Fisher Scientific, Loughbrough, UK) to apply shear stress and incubated for variable required time at 37 °C and 50 rpm.

The transcriptional response of the cells to fluid shear stress in a two-armed study (mechanically strained vs. static cells) was carried out to rule out factors other than mechanical stress affecting the osteogenic response of the cells. The magnitude of mechanical strain applied was restricted to 10–12 dynes/cm^2^ and calculated using the formula τ = a(ηρ(2πf)3)1/2, where a is the orbital radius of rotation of the shaker, ρ is the density of the culture medium (0.9973 g/mL), η is the viscosity of the medium (0.0101) and f is the frequency of rotation (rotation/s). Following the application of mechanical strain, the expression of genes associated with osteogenic differentiation (*ALP*, *RUNX2* and *COL1A1*) and genes of interest that might play a role in mediating the effect of FSS (*TAZ*, *GLI3* and *ZIC1*) were determined.

The immunostaining intensity (i.e., *ZIC1* and *RUNX2*) was measured for a total of 20 cells per treatment using ImageJ 1.42q software (U.S. National Institutes of Health, Bethesda, MD, USA), for a total of 20 cells per treatment. A straight line of a of distance of 100 pixels was drawn along the long axis of each cell such that it passed through the middle of the nucleus and passed through the cytoplasm at the start and at the end. For each pixel for a given cell, the colour intensity was given as mean and SD, and it was plotted on a graph against the *x*-axis as the pixel distance.

### 4.12. Quantitative RT-PCR

Validation of the Affymetrix gene expression data was carried out for selected transcripts using TaqMan gene expression assays and the Applied Biosystems Prism 7900 HT sequence detection system. From each donor, total RNA (500 ng) was reverse-transcribed (Omniscript, Qiagen Ltd.), and resulting cDNA, which represented 2.5 ng total RNA, was the used in the PCR reaction. The analyses were performed in duplicate. The gene expression results are given as RQ (relative quantitation), and the relative changes of a given gene transcript were calculated using the 2(ΔΔC(T)) method. The ribosomal protein L41 (RPL41) served as an endogenous control; this was selected as internal standard because Affymetrix analyses had shown similar signal values in all samples (10,895 ± SD 315).

## 5. Conclusions

This study unravels the molecular sensory machinery and downstream effector molecules in osteoblasts and osteocytes, identifying *ZIC1* as an important anabolic factor to induce bone formation during mechanical stress and microfractures. The present results suggest the clinical relevance of *ZIC1* as a mechanosensory regulator to modulate osteogenic anabolic activity, which, after further study, may provide a potential therapeutic target.

## Figures and Tables

**Table 1:** (**A**) Comparison of *ZIC1* promoter methylation in trabecular bone biopsies from male iliac crest (IB) and the lumbar spine (L5), as well as the correlation between the degree of *ZIC1* promoter methylation at CpG positions and *ZIC1* expression. (**B**) Differential expression of *ZIC1*. The mean relative expression levels of *ZIC1* mRNA in the lumbar spine vertebrae and iliac crest trabecular bone from men.

(A)																				
*ZIC1* Methylation Site	Pos. 1	Pos. 2	Pos. 3	Pos. 4	Pos. 5	Pos. 6	Pos. 7	Pos. 8	Pos. 9	Pos. 10	Pos. 11	Pos. 12	Pos. 13	Pos. 14	Pos. 15	Pos. 17	Pos. 18	Pos. 19	Pos. 20	Mean
**Mean IC** **(*n* = 7)**	49.2	23.7	25	16.2	23.5	18.6	19.9	11.7	11.3	24.5	12.5	9.4	13.8	16.1	9.5	9.9	8.3	5	8.8	16.7
**Mean L5** **(*n* = 7)**	31.8	15.4	18.5	11.7	15.3	12.6	12.7	7.5	7.5	16.8	8.6	6.6	10.9	11	6.3	6.9	6	4.1	5.3	11.3
***p*** **-value** **(L5 vs. IB)**	9 × 10−^4^	0.003	0.006	0.009	0.005	0.009	0.006	0.011	0.01	0.01	0.015	0.011	0.042	0.007	0.004	0.004	0.019	0.055	0.032	0.004
**Pearson *r* (ZIC1 vs. methylation)**	−0.719	−0.71	−0.56	−0.47	−0.71	−0.64	−0.74	−0.56	−0.59	−0.72	−0.62	−0.455	−0.409	−0.677	−0.682	−0.316	−0.346	−0.327	−0.343	−0.67
(**B**)																				
***ZIC1*** **Probe set ID**	**Iliac Crest**				**L3**				**L4**				**L5**				**ANOVA *p*-Value**			
206373_at	18				865				285				856				5.56 × 10^−10^			
234716_at	37				39				39				52				0.853			
236896_at	8				18				14				23				0.0003			

**Table 2:** Correlation between *ZIC1* expression level in iliac bone of postmenopausal women with BMD, lean body and fat mass (*n* = 57).

	Pearson *r*	*p*-Value
Total hip Z-score, BMI adj	−0.383	0.003
Total hip Z-score	−0.369	0.005
Femoral neck Z-score, BMI adj	−0.331	0.012
Femoral neck Z-score	−0.323	0.011
L1–L4 Z-score, BMI adj	−0.337	0.01
L1–L4 Z-score *	−0.337	0.01
Total body Z-score, BMI adj *	−0.398	0.003
Total body Z-score	−0.395	0.004
Lean mass (g)	0.118	NS
Fat mass (g)	0.108	NS

Probe set for *ZIC1* expression level: 206373_at * *n* = 51.

**Table 3:** Differential *ZIC1* DNA methylation levels observed between osteoporotic (OP) and healthy subjects at iliac bone site.

I.D.	Mean Healthy (*n* = 40)	±SD	Mean OP (*n* = 29)	±SD	*p*-Value
cg17280346	0.24	0.04	0.30	0.07	0.0004 *
cg01227537	0.14	0.03	0.17	0.06	0.0043
cg16636671	0.11	0.03	0.14	0.04	0.0116
cg23449696	0.34	0.04	0.37	0.04	0.0138
cg05716671	0.10	0.02	0.11	0.03	0.0211
cg15002294	0.07	0.01	0.08	0.03	0.0224
cg05371578	0.19	0.03	0.21	0.05	0.0257
cg02519751	0.19	0.04	0.23	0.08	0.0294
cg14750948	0.19	0.04	0.22	0.06	0.0344
cg16209664	0.18	0.03	0.19	0.04	0.0413
cg14456683	0.26	0.03	0.28	0.05	0.0480
cg19029181	0.25	0.06	0.28	0.06	0.0499

This table shows CpGs with DNA methylation levels differing between OP and healthy (nominally significant). *p*-values were calculated using Student’s *t*-test. * Shows significance after adjustment for multiple testing (*p* = 0.00041* 20 *ZIC1* CpGs = 0.008).

**Table 4:** (**A**) Comparison between *ZIC1* promoter DNA methylation at five positions in primary human skin fibroblasts (HSFs) and osteoblast-like human osteosarcoma cell line (SAOS2 cells). (**B**) Effect of FSS on modulation of gene transcription/expression in osteoblast-like human osteosarcoma cell line (SaOS2 cells). (**C**) Effect of FSS on transcription in primary human skin fibroblasts (HSFs).

(A)								
Cell Type		Pos. 1		Pos. 2	Pos. 3		Pos. 4	Pos. 5
HSFs (Mean ± SD, *n* = 3)		43.99 ± 3.62		8.89 ± 5.03	26.72 ± 12.42		11.73 ± 8.79	6.57 ± 3.54
SAOS (Mean ± SD, *n* = 3)		3.34 ± 0.62		1.88 ± 0.16	2.55 ± 1.12		1.15 ± 0.147	0.96 ± 0.38
**(B)**								
**Gene**	**Static** **(Delta Ct Mean ± SD)** **(*n* = 3)**		**FSS** **(Delta Ct Mean ± SD)** **(*n* = 3)**			**Fold Change (ln)** **FSS vs. Static**		***p*** **-Value**
*RUNX2*	3.787 ± 0.081		3.622 ± 0.011			1.12		0.0049
*ZIC1*	6.983 ± 0.034		6.694 ± 0.096			1.22		NS
*SP7*	6.088 ± 0.089		5.517 ± 0.058			1.49		0.0003
*KIF3A*	6.877 ± 0.037		6.608 ± 0.085			1.20		0.0045
*GLI3*	6.000 ± 0.009		5.979 ± 0.111			1.01		NS
*GLI2*	7.240 ± 0.008		7.166 ± 0.054			1.05		NS
*GLI1*	9.617 ± 0.107		9.205 ± 0.169			1.33		0.0176
*PTCH1*	6.878 ± 0.017		6.890 ± 0.170			−1.01		NS
*COL1A1*	−1.645 ± 0.056		−1.571 ± 0.174			−1.05		NS
*ALPL*	0.530 ± 0.072		0.546 ± 0.209			−1.01		NS
*SMO*	6.295 ± 0.017		6.369 ± 0.238			−1.05		NS
**(C)**								
**Gene**	**Static** **(Delta Ct Mean ± SD)** **(*n* = 3)**		**FSS** **(Delta Ct Mean ± SD)** **(*n* = 3)**			**Fold Change (ln)** **FSS vs. Static**		***p*** **-Value**
*RUNX2*	7.650 ± 0.266		7.341 ± 0.227			1.14		NS
*ZIC1*	10.391 ± 0.227		10.204 ± 0.192			1.22		NS
*SP7*	12.455 ± 0.452		12.331 ± 0.943			1.09		NS
*KIF3A*	7.252 ± 0.106		6.750 ± 0.026			1.42		0.0006
*GLI3*	6.7434 ± 0.155		6.410 ± 0.045			1.26		0.0174
*GLI2*	6.966 ± 0.245		6.624 ± 0.022			1.27		NS
*GLI1*	12.1314 ± 0.229		11.181 ± 0.323			1.93		0.0098
*PTCH1*	9.602 ± 0.144		9.597 ± 0.094			1.0		NS
*COL1A1*	−3.255 ± 0.189		−3.0435 ± 0.192			−1.16		NS
*ALPL*	13.475 ± 0.585		11.475 ± 0135			−4.00		0.0025
*SMO*	8.121 ± 0.165		7.666 ± 0.119			−1.37		0.0128

Numbers indicate methylation levels expressed as percentage (%).

## Data Availability

The data for postmenopausal biopsies have been submitted to the European Bioinformatics Institute (EMBL-EBI; ArrayExpress repository, ID: E-MEXP-1618). The data on male biopsies are accessible through accession number E-MEXP-2219. The scripts for analysis are available at https://github.com/Bioinformatics-Support-Unit/python-scripts/tree/master/zic1 (accessed on 26 April 2016).

## Supplementary Materials

The following supporting information can be downloaded at: https://www.mdpi.com/article/10.3390/ijms23062957/s1.
