# Supplementary material for: Mechanical-Stress-Related Epigenetic Regulation of ZIC1 Transcription Factor in the Etiology of Postmenopausal Osteoporosis
Source: Int J Mol Sci. 2022 Mar 9;23(6):2957. doi: 10.3390/ijms23062957 (PMC8955993; doi:10.3390/ijms23062957)
Supplement: Supplementary file 1 [file ijms-23-02957-s001.zip › ijms-1564866-supplementary.pdf]

Supplementary Material

Mechanical-Stress-Related Epigenetic Regulation of *ZIC1* Transcription Factor in the Etiology of Postmenopausal Osteoporosis

Biochemical parameters

Urinary type I collagen N-terminal telopeptide (U-NTx) was measured using Osteomark Urine NTx kit (Wampole Laboratories Inc., Princeton, NJ, USA), as an indicator of bone resorption, with intra- and inter assay CVs of 8% and 15%, respectively. The normal range for the age group 40–58 is <131 mM. Urine Pyrilinks-D (i.e., U-DPD) was measured using Immulite 2500 (Siemens Healthcare Diagnostics Inc., Erlangen-Höchstadt, Germany). The inter and intra CVs were 9–18% and 5.9–15%, respectively, with normal range 3.0–13.6 mM deoxypyridinium crosslinks (DPYP)/mM creatinin (for subjects >55 years old). Levels of alkaline phosphatase (ALP), type I collagen C-terminal telopeptide (1-CTP), and vitamin D were analyzed as described [1]. All blood parameters related to kidney, heart, liver, general endocrine and malabsorption status were normal.

Subject evaluation and exclusion criteria

Subjects

The research participations were Caucasians men from Tyne and Wear County, U.K., and undergoing lumber spinal orthopedic surgery, without any evidence of recent or past history of osteoporosis related fractures. To avoid inconsistencies in taking LS and IB bone biopsies all the surgical procedures and biopsies were carried out by one surgeon.

In additional to routine laboratory investigation, other possible secondary causes of osteoporosis were excluded by detail medical history and examination. The following questionnaire was used:

Family History

Family history of osteoporosis and osteoporosis-related fractures without trauma were excluded.

Social History

The questions related to alcohol intake, smoking, excessive caffeine intake, lack of exposure to sunlight, malnutrition, past history of exposure to heavy metals (such as workers in shipyards), particularly low body weight and frailty.

Medical History

The history specifically excluded medical history of anorexia, weight loss, height loss, GI disorders, neoplastic disease, prolonged immobilization or poor physical activity, hypercalciuria, endocrine diseases, connective tissue disorders, renal disorders, past history of fracture as an adult.

Medication history

The subjects on chronic treatment with steroids, excessive thyroid hormone, anticoagulant, anticonvulsant, lithium, immunosuppressive drugs were excluded.

Microarray data pre-processing and evaluation

Differentially expressed genes were identified using the Bayesian ANOVA for microarrays (BAMarray) method (www.bamarray.com). This method takes the problem of multiple testing into account by controlling not only the false

discovery rate (FDR) but also the false non discovery rate (FNR), true transcript changes that are not discovered. For identifying differentially expressed genes, a Bayesian test statistic is estimated for each gene.

The osteoporosis and control groups were not balanced with respect to age and BMI. Therefore, for each differentially expressed gene, we examined to what extent its gene expression was explained by disease, age, and/or BMI. We used the R-package Limma (Linear Models for Microarray Data), which is particularly useful for the analysis of linear models in designed experiments.[2] First, a linear model was fitted for each gene (using the Limma function lmFit). Then moderated t and F statistics were computed for each gene and contrast, that is, disease status, age, and BMI, by empirical Bayes shrinkage of the standard errors toward a common value (using the Limma function ebayes). Finally, for each gene that was identified as significantly differentially expressed using BAMarray, we tested whether its expression was explained by disease, age, and/or BMI using the Limma function classifyTestsF. This function uses a nested F-test approach where p values are adjusted for testing across the contrasts, that is, disease status, age, and BMI, but not for testing across the genes.

We eliminated probe sets containing more than 43 absent calls (according to the Affymetrix MAS 5.0 software) in the cohort across the entire data set, resulting in a reduction of informative probe sets from 54,675 to 22,815. The data were normalized using the PLIER (Probe Logarithmic Intensity Error) algorithm in Array Assist to calculate relative signal values for each probe set. In order to filter out low signal values, the MAS5 algorithm in Array Assist was used to create an Absolute Call dataset showing the number of present and absent calls for each probe set. The correlation (Pearson) was computed between expression of each gene and the BMD across 84 women using log transformed signal values. Zero correlation was tested for each gene against the two-tailed hypothesis.

The overview of differentially expressed genes in the LS relative to IB in male controls was generated by the use of Affymetrix software, which made it possible to compare data from two arrays. The statistical analysis is based on 22 different cDNA oligonucleotides to measure quantitatively one mRNA transcript, and each cDNA probe is distributed as 22 different "micro-spots". Thus, 22 signals for each mRNA transcript (probeset) are generated and enables the Affymetrix software GCOS to compute p-values for differential expression when two transcripts on two different arrays are compared. The Wilcoxon's Signed Rank test uses the differences between Perfect Match and Mismatch probe signal intensities, as well as the differences between Perfect Match intensities and background to compute each p-value difference. From Wilcoxon's Signed Rank test, a total of three, one-sided p-values are computed for each probe set. The most conservative value is chosen to determine the "change call". That is the value closest to 0.5 signifying that no change is detected. These are combined to give one final p-value.

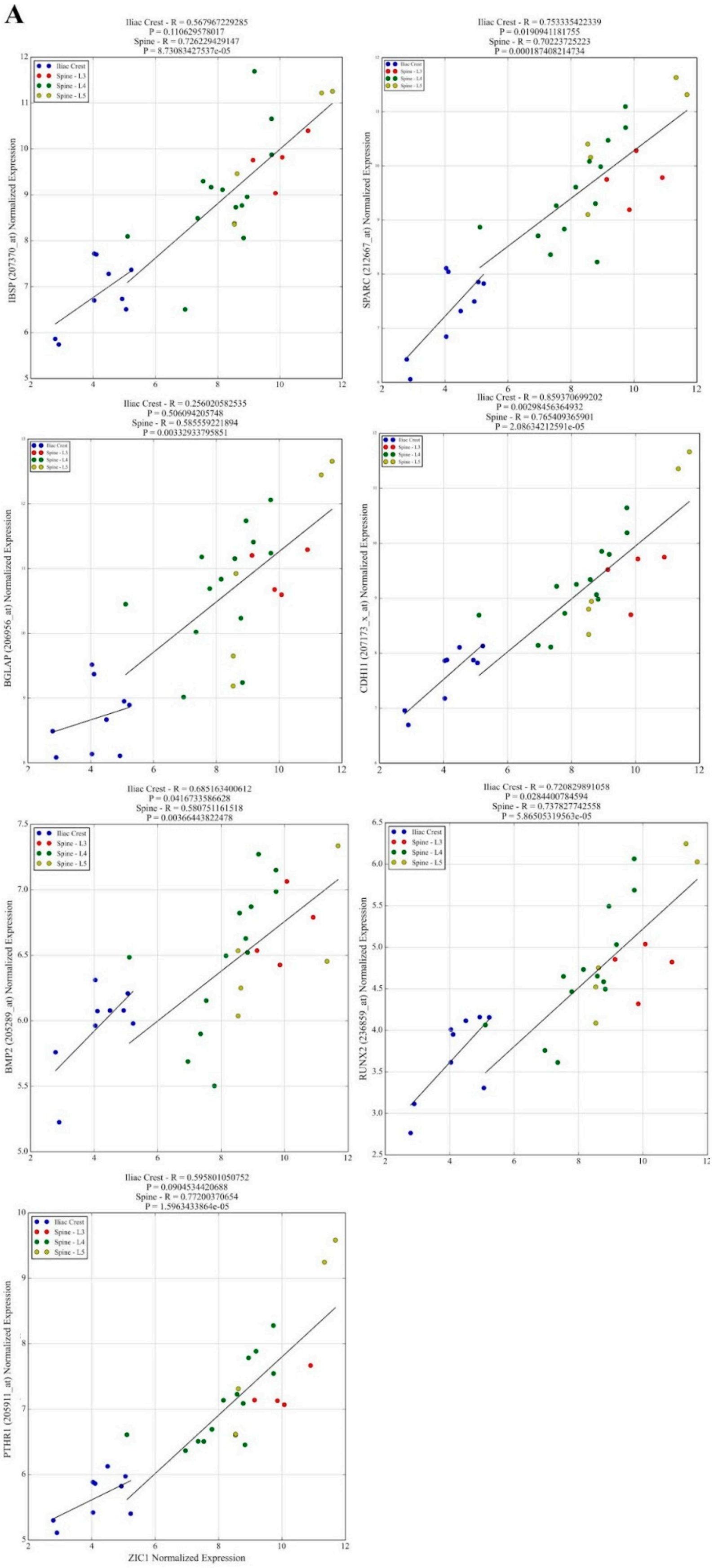

Figure S1. *Cont.*

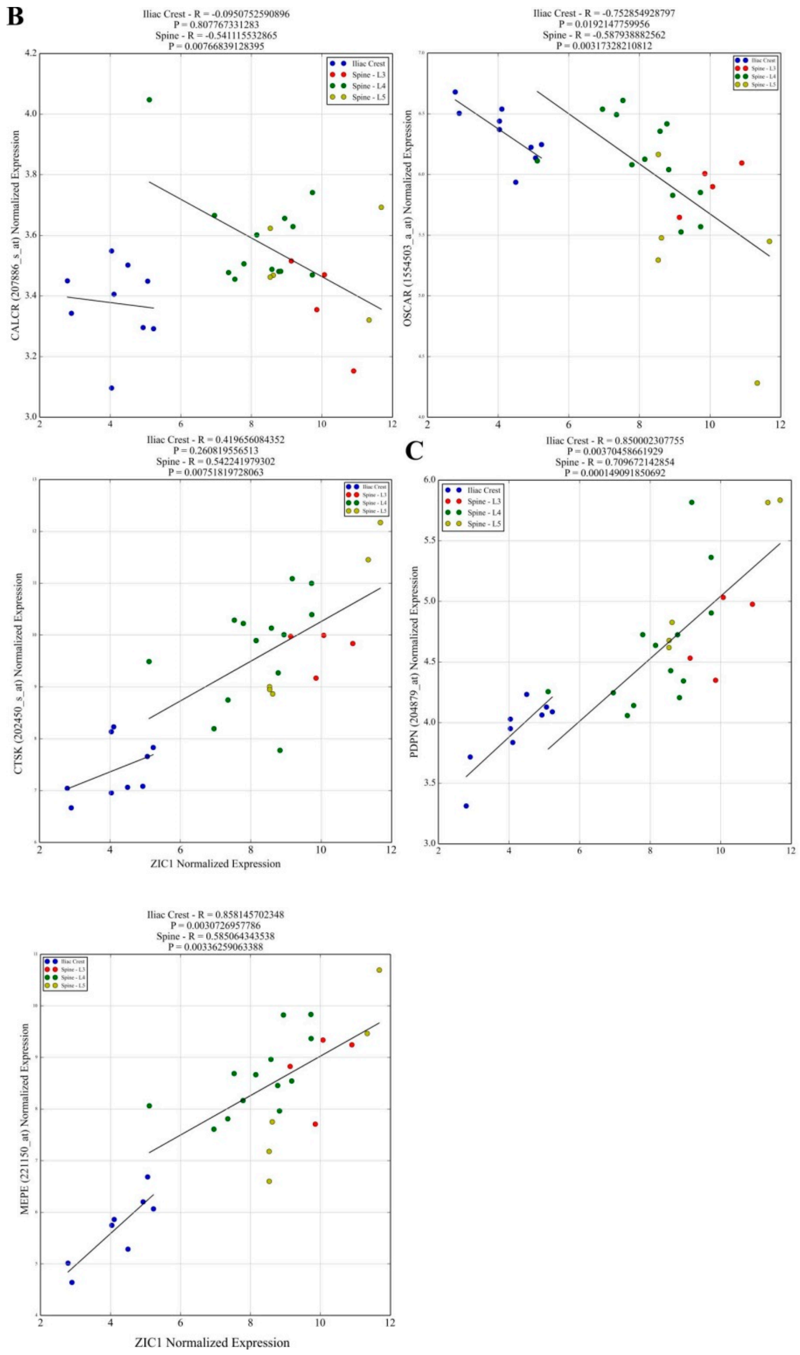

Figure S1. *ZIC1* expression levels correlated with the transcripts reflecting osteoblast matrix forming activity. (**A**) Transcripts for *IBSP*, *SPARC*, *BGLAP*, *CDH11* and osteoblast differentiation (*BMP2*, *RUNX2)* showed strong positive correlation with *ZIC1* in the lumbar spine, however had weaker or no correlation in IB. The *PTHR1* transcript used to indicate osteoblast number also showed a strong positive correlation with *ZIC1* expression. (**B**) *CTSK* transcripts that reflect osteoclast activity showed strong positive correlation with *ZIC1* expression. Osteoclast associated transcripts *CALCR* and *OSCAR* showed inverse correlation with *ZIC1* expression in the lumbar spine but weaker and no correlation respectively in the iliacus, probably due to fewer samples. (**C**) Osteocyte associated transcripts *PDPN* and *MEPE* expression at both high (LS) and low (IB) bone turnover sites showed strong correlation with *ZIC1* mRNA.

Table S1. Demographic, clinical and laboratory parameters of the cohort of men.

	Mean (*n* = 13)	SD	Laboratory Reference Ranges
Age (years)	52.07	11.82	NA
BMI (kg/m^2)	29.86	3.18	NA
PTH (ng/L)	37.6	15.1	10-60
Calcium (mmol/L)	2.38	0.14	2.20–2.65
ALP (bone specific) (u/L)	58	9.42	30-125
Phosphate (mmol/L)	1.18	0.15	0.85–1.50
BMD, spine (L1–L4, g/cm^2)	1.06	0.17	NA
BMD, hip (total, g/cm^2)	1.05	0.15	NA
BMD, neck of hip (g/cm^2)	0.87	0.15	NA
Smoking (years)	0	0	NA

Table S2. Demographic, clinical and laboratory parameters of the postmenopausal women with a range of BMD.

	Mean (*n* = 57)	SD	Laboratory Reference Ranges
Age (years)	64.56	9.57	NA
BMI (kg/m^2)	24.17	3.63	NA
Vitamin K (µg/L)	0.53	0.31	0.1–3.0
PTH (pmol/ L)	4.45	2.11	1.10–7.70
Ca2+ corr. (mmol/L)	1.25	0.04	1.15–1.35
25(OH)vitD (nmol/L)	83.43	34.63	30–110
Osteocalcin (nmol/L)	1.47	0.61	4–12
1CTP (µg/L)	4	1.48	1.8–5.0
ALP (bone specific) (U/L)	24.68	9.42	11.6–30.6
Phosphate (mmol/L)	1.18	0.15	0.85–1.50
BMD, spine (L1–L4, g/cm^2)	0.86	0.34	NA
BMD, hip (total, g/cm^2)	0.89	0.19	NA
BMD, neck of hip (g/cm^2)	0.86	0.17	NA
Number of given births	1.8	1.19	NA
Smoking (years)	13.2	17.57	NA

Table S3. Clinical Characteristics and Laboratory Data of Osteoporotic Patients (OP) and Controls (CTR).

	CTR	OP	*p*-Value
Bone biopsies (*n*)	40	29	
Age (years)	62.2 ± 8.2	69.4 ± 10.60	0.005
BMI (kg/m^2)	25.6 ± 3.9	22.6 ± 3.04	0.001
Serum vitamin K (µg/L)	0.51 ± 0.24	0.46 ± 0.23	0.58
Serum intact PTH (pmol/L)	4.2 ± 1.9	5.1 ± 2.5	0.09
Serum Ca^{2+} corr. (mmol/L)	1.24 ± 0.04	1.25 ± 0.05	0.58
Serum $25(OH)_2D_3$ (nmol/L)	80.4 ± 37.4	87.8 ± 44.0	0.49
Bone markers			
Serum osteocalcin (nmol/mL)	1.34 ± 0.54	1.57 ± 0.67	0.15
Serum bone-specific ALP (U/L)	21.0 ± 8.3	29.0 ± 9.4	0.001
Urinary NTX (mmol/L)	53.4 ± 25.0	76.2 ± 48.0	0.036
Serum 1-CTP (CTX) (µg/L)	3.64 ± 0.97	4.67 ± 2.12	0.025
Urinary DPD (mM DPD/mM Cr)	7.01 ± 2.55	7.80 ± 2.29	0.205
Serum phosphate (mmol/L)	1.18 ± 0.16	1.18 ± 0.15	0.88
BMD (T-score)			
Total hip	0.51 ± 0.79	-2.66 ± 0.78	*
Hip neck (mean)	0.17 ± 0.67	-2.61 ± 0.53	*
LS (L_2–L_4)	0.56 ± 0.85	-3.42 ± 0.94	*

LS (L_1)	0.18 ± 0.76	-3.48 ± 0.96	*
BMD (g/cm²)			
Total body	1.20 ± 0.06	0.93 ± 0.07	*
Total hip	1.06 ± 0.09	0.68 ± 0.09	*
Hip neck (mean)	1.00 ± 0.08	0.68 ± 0.08	*
LS (L_2–L_4)	1.29 ± 0.08	0.77 ± 0.12	*
LS (L_1)	1.15 ± 0.10	0.72 ± 0.12	*
BMD (Z-score)			
Total body	1.63 ± 0.92	-1.09 ± 0.82	*
Total hip	1.30 ± 0.94	-1.27 ± 0.62	*
Hip neck (mean)	1.18 ± 0.79	-1.00 ± 0.44	*
LS (L_2–L_4)	1.68 ± 0.99	-1.97 ± 0.82	*
LS (L_1)	1.14 ± 1.00	-1.75 ± 0.84	*

Data are expressed as mean ± SD. Bone mineral density (BMD) data are expressed either as *T*-scores (g/cm²) or BMD *Z*-scores (age-adjusted *T*-score values given with reference to normative data provided by the instrument manufacturer). * $p < .0001$ (independent samples *t*-test). $p < 0.05$ considered significant (light gray shading: $p < 0.05$; dark gray shading: $p \leq 0.001$).

Table S4. Clinical characteristics and laboratory data of the validation group (VG).

	VG	*p*-Values	
		VG vs. OP	VG vs. CTR
Bone biopsies (*n*)	18	n.a.	n.a.
Age (years)	62.4 ± 8.3	0.022	0.951
BMI (kg/m²)	23.5 ± 2.7	0.270	0.051
No. of smokers in each group	6	n.a.	n.a.
Smoking (years)	8.8 ± 14.1	0.052	0.807
No. of patients taking supplement HRT	5	n.a.	n.a.
E2 (years)	1.83 ± 3.9	0.428	0.052
S-Vitamin K (μg/L)	0.61 ± 0.44	0.304	0.334
S-intact PTH (pmol/L)	4.0 ± 1.58	0.107	0.822
S-Ca^{2+} corr. (mmol/L)	1.27 ± 0.04	0.157	0.037
S-25(OH)D3 (nmol/L)	92.3 ± 32.4	0.711	0.056
Bone markers			
S-Osteocalcin (nmol/ml)	1.51 ± 0.66	0.587	0.306
S-bone specific ALP (U/L)	26.60 ± 8.92	0.409	0.024
U-NTX (mmol/L)	67.71 ± 34.86	0.989	0.060
S-1-CTP (CTx) (μg/L)	3.79	0.103	0.584
U-DPD (mM DPD/mM Cr)	7.59 ± 1.97	0.858	0.319
S-phosphate (mmol/L)	1.17 ± 0.16	0.913	0.721
BMD (*T* score)			
Total hip	−1.24 ± 0.71	*	*
Hip neck (mean)	−1.34 ± 0.69	*	*
LS (L2–L4)	−3.37 ± 0.52	0.061	*
LS (L1)	−2.96 ± 0.76	0.128	*
BMD (g/cm²)			
Total Hip	0.85 ± 0.09	*	*
Hip neck (mean)	0.83 ± 0.09	*	*
LS (L2–L4)	0.83 ± 0.06	0.085	*
LS (L1)	0.78 ± 0.09	0.114	*
BMD (*Z* score)			

Total body	−0.41±0.79	0.012 *	*
Total Hip	−0.28±0.78	*	*
Hip neck (mean)	−0.14±0.65	*	*
LS (L2–L4)	−1.98±0.69	0.956	*
LS (L1)	−1.76±0.76	0.970	*

Data are expressed as mean ± SD. Bone mineral density data are expressed either as BMD (g/cm^2), BMD *T*-scores (the number of standard deviations (SD) from the mean BMD for young adult women), or BMD *Z*-scores (age-adjusted *T*-score values). * $p < 0.001$ (independent samples *t*-test) (dark shaded). $p < 0.05$ (light shaded) considered significant. n.a., not applicable.

Reference

1. Jemtland, R.; Holden, M.; Reppe, S.; Olstad, O.K.; Reinholt, F.P.; Gautvik, V.T.; Refvem, H.; Frigessi, A.; Houston, B.; Gautvik, K.M. Molecular disease map of bone characterizing the postmenopausal osteoporosis phenotype. *J. Bone Miner. Res.* **2011**, *26*, 1793–1801.
